# Patient-reported outcomes in neuro-oncology

**DOI:** 10.1097/CCO.0000000000001078

**Published:** 2024-07-10

**Authors:** Josien C.C. Scheepens, Martin J.B. Taphoorn, Johan A.F. Koekkoek

**Affiliations:** Department of Neurology, Leiden University Medical Center, Leiden, the Netherlands

**Keywords:** brain tumor, functioning, health-related quality of life, patient-reported outcome, patient-reported outcome measures, symptom

## Abstract

**Purpose of review:**

To provide up-to-date evidence on patient-reported outcomes (PROs) in neuro-oncology, with a focus on the core constructs of health-related quality of life (HRQoL) and the use of PROs in clinical trials and clinical practice.

[Supplemental Digital Content: Video Abstract PROs in Neuro-Oncology.mov]

**Recent findings:**

PROs are gaining importance in brain tumor research and medical care. For patients with a brain tumor, core PRO constructs are pain, difficulty communicating, perceived cognition, seizures, symptomatic adverse events, physical functioning and role and social functioning, which are assessed through patient-reported outcome measures (PROMs). Initiatives have been taken to improve the reliability and robustness of PRO data, including standardization of items included in clinical trial protocols (the SPIRIT-PRO extension) and formulation of PRO priority objectives for use in clinical trials (the SISAQOL-Innovative Medicines Initiative). In brain tumor patients with cognitive impairment, caregiver-reported outcomes may complement or replace PROs to increase accuracy. The next key challenge will be to widely implement PROs and apply PRO data in clinical practice to benefit patients with brain tumors.

**Summary:**

PROs are clinically relevant endpoints providing information only known by the patient. Standardization of the use of PROs in clinical trials and wide implementation in clinical practice is needed to improve HRQoL of brain tumor patients.

## INTRODUCTION

Clinical outcomes to measure treatment effect in adult patients with a brain tumor historically included ‘objective’ endpoints such as overall survival, progression-free survival, and radiological response on imaging. However, as patients with a brain tumor may have a limited life expectancy and suffer from neurological, cognitive, and general symptoms caused by the tumor or by treatment, a patient-centered outcome measurement approach is pivotal for brain tumor patients. In this light, Clinical Outcome Assessments (COAs), which measure a patient's symptoms, mood, or the effects of a disease or condition on the patient's level of functioning, have gained more attention over the past decades [1]. The four types of COAs include patient-reported outcomes (PROs), clinician-reported, and caregiver-reported outcomes and performance outcomes [1]. In this review article, recent literature on PROs in patients with primary and secondary brain tumors will be discussed. 

**Box 1:**
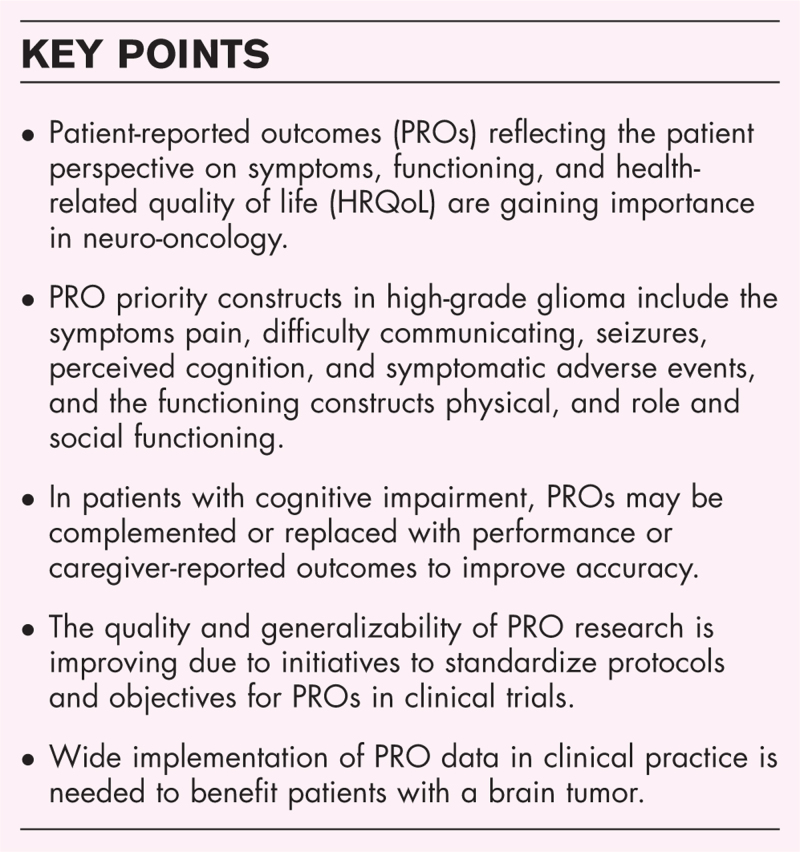
no caption available

## PATIENT-REPORTED OUTCOMES AND PATIENT-REPORTED OUTCOME MEASURES

PROs reflect the status of a patient's health condition, which can be quantified using patient-reported outcome measures (PROMs) without amendment or interpretation of the response by a clinician or anyone else [2]. A lot of heterogeneity exists in the type of PROMs used in brain tumor studies to date, as 215 different PROMs have been identified in the literature, of which 70% were only used once or twice [3]. To standardize the use of PROs, in 2020 the Fast Track working group from the Response Assessment in Neuro-Oncology (RANO-) PRO agreed on a core set of PROs to be measured in high-grade glioma clinical trials and practice, which are all considered components of health-related quality of life (HRQoL). These include five symptom constructs (pain, difficulty communicating, perceived cognition, seizures, and symptomatic adverse events) and two functioning constructs (physical and role functioning, of which the latter also incorporates social functioning) [4]. PROMs may be unidimensional, that is, measuring a single construct such as the Seizure Severity Questionnaire, or multidimensional, measuring multiple constructs such as the European Organisation for Research and Treatment of Cancer (EORTC) Quality of Life Questionnaire - Core 30 (QLQ-C30). Table 1 summarizes how the priority constructs are included in the most frequently used multidimensional PROMs in brain tumor patients. There has been debate on to what extent to rely on PROs in brain tumor patients, especially considering the high prevalence of cognitive deficits in this population. In the next paragraphs, we will discuss recent literature on the core PRO constructs, and review the application and limitations of PROs and PROMs in neuro-oncology clinical trials and clinical practice.

**Table 1:** Frequently used multidimensional patient-reported outcome measures and incorporated priority constructs^a^

PROM	Full name	Population of intended use	Time period of interest	Subscales (total # of items)	Total # of items (# of priority items)	Priority construct(s) (# of priority items)	Specific items per construct (with corresponding number in PROM)
EORTC QLQ-C30	European Organization for Research and Treatment of Cancer Quality of Life Questionnaire – Core 30	Cancer-specific	Past week	Global health/Quality of life (2) Physical functioning (5) Role functioning (2) Emotional functioning (4) Cognitive functioning (2) Social functioning (2) Single items (13)	30 (13)	Physical functioning (5) Role and social functioning (4) Perceived cognition (2) Pain (2)	Physical functioning: *vigorous activity (1); walking (2, 3); bed/chair (4); ADL (5)* Role and social functioning: *work (6); hobbies (7); family (26); social (27)* Perceived cognition: *concentration (20); memory (25)* Pain: *(9); interference (19)*
EORTC QLQ-BN20	European Organization for Research and Treatment of Cancer Quality of Life Questionnaire – Brain Cancer 20	Brain tumor-specific	Past week	Future uncertainty (3) Visual disorder (3) Motor dysfunction (3) Communication deficit (3) Single items (7)	20 (9)	Difficulty communicating (4) Physical functioning (2) Role and social functioning (1) Pain (1) Seizures (1)	Difficulty communicating: *speech (41, 42, 43), reading (38)* Physical functioning: *decline (32), weakness limbs (48)* Role and social functioning: *family (33)* Pain: *headache (34)* Seizures: *(39)*
FACT-Br	Functional Assessment of Cancer Therapy - Brain	Brain tumor-specific	Past week	Physical well being (7) Social/family well being (7) Emotional well being (6) Functional well being (7) Additional concerns (23)	50 (24)	Physical functioning (4) Role and social functioning (10) Pain (2) Symptomatic adverse effects (1) Difficulty communicating (3) Perceived cognition (4)	Physical functioning: *meeting needs (GP3), in bed (GP7), ADL (Br14); weakness limbs (Br20)* Role and social functioning: *friends (GS1, GS3), family (GS2, GS4, GS5), partner (GS6), sex (GS7), work (GF1, GF2), driving (Br18);* Pain: *(GP4); headache (An10)* Symptomatic adverse effects: *(GP5)* Difficulty communicating: *speech (Br8, Br9); reading (Br16), writing (Br17)* Perceived cognition: *memory (Br3); thinking (Br13); deciding (Br11); acting (Br15)*
SF-36	Short Form 36 Health Survey	Generic	Past 4 weeks	Physical functioning (10) Role limitations due to physical health (4) Role limitations due to emotional problems (3) Energy/fatigue (4) Emotional well being (5) Social functioning (2) Pain (2) General health (5)	36 (21)	Physical functioning (10) Role and social functioning (9) Pain (2)	Physical functioning: *activity (3–5, 8); stairs (6, 7); walking (9–11); ADL (12)* Role and social functioning: *physical interference (13–16); emotional interference (17–19); social (20, 32)* Pain: *(21); interference (22)*
MDASI-BT	MD Anderson Symptom Inventory Brain Tumor	Brain tumor-specific	Past day	Symptom severity (22) Symptom interference (6)	28 (11)	Physical functioning (3) Role and social functioning (2) Pain (1) Perceived cognition (2) Communication difficulty (2) Seizures (1)	Physical functioning: *activity (23), walking (27), weakness limbs (14)* Role and social functioning: *work (25), social (26)* Pain: *(1)* Perceived cognition: *memory (7), concentration (18)* Communication difficulty: *understanding (15), speaking (16)* Seizures: *(17)*
EORTC QLQ-C15-PAL	European Organization for Research and Treatment of Cancer Quality of Life Questionnaire - Core 15 - Palliative Care	Cancer-specific	Past week	Physical functioning (3) Emotional functioning (2) Fatigue (2) Pain (2) Single items (5) Overall quality of life (1)	15 (5)	Physical functioning (3) Pain (2)	Physical functioning: *walk (1); bed/chair (2); ADL (3)* Pain: *(5), interference (12)*
FACT-G	Functional Assessment of Cancer Therapy - General	Cancer-specific	Past week	Physical well being (7) Social/family well being (7) Emotional well being (6) Functional well being (7)	27 (13)	Physical functioning (2) Role and social functioning (9) Pain (1) Symptomatic adverse effects (1)	Physical functioning: *meeting needs (GP3); bed (GP7)* Role and social functioning: *friends (GS1, GS3), family (GS2, GS4, GS5), partner (GS6), sex (GS7), work (GF1, GF2)* Pain: *(GP4)* Symptomatic adverse effects: *(GP5)*
QOLIE-31-P	Patient Weighted Quality of Life in Epilepsy	Epilepsy-specific	Past week	Overall quality of life (1) Part A: feeling (5) Part B: feeling (6) Part C: activities (6) Part D: cognition (7) Part E: epilepsy and ASM (4) Part F: feeling about seizures (6) Part G: feeling overall quality of life (2) Part H: health (1) Part I: areas of importance (1)	39 (23)	Role and social functioning (6) Perceived cognition (7) Symptomatic adverse effects (4) Seizures (6)	Role and social functioning: *social (13, 17); hobbies (14), driving (15), work (16); distress (18)* Perceived cognition: *reasoning (19); memory (20, 21, 24); concentrating (22, 23), distress (25)* Symptomatic adverse effects: *(26–28), distress (29)* Seizures: *worry (30–33), bother (34), distress (35)*
SQLI	Spitzer Quality of Life Index	Cancer-specific	Past week	Single items (5)	5 (3)	Physical functioning (1) Role and social functioning (2)	Physical functioning: *ADL/transport (1)* Role and social functioning: *work (2), social (4)*

a Frequently used multidimensional PROMs in brain tumor patients as identified by Dirven *et al.*[3].

## HEALTH-RELATED QUALITY OF LIFE

HRQoL is a multidimensional construct that comprises the patient's perceptions of disease symptoms, physical, emotional, social, and cognitive functions, and side effects of treatment [5]. Living with a brain tumor may have a large impact on a patient's HRQoL. As patients’ mean age, tumor grade, treatment, and prognosis vary between tumor types, the extent of HRQoL impairment may also differ. Patients with a low-grade glioma have a significantly better HRQoL than high-grade glioma patients, though worse than meningioma patients, whose HRQoL is also lower than the HRQoL of healthy controls [6^▪^,^7^]. Toxicity of treatment, including cerebral radiation necrosis or cognitive deficits after chemotherapy or radiotherapy, may pose a high burden on brain tumor patients on the long term [8^▪^]. For example, low-grade glioma patients show impairments in multiple HRQoL domains multiple years after treatment [9]. Also, survivorship issues, such as uncertainty about the disease, its emotional impact, and challenges to maintain a social life, may compromise patients’ HRQoL [10^▪^]. The most commonly used PROMs in brain tumor patients to quantify HRQoL are the EORTC QLQ-C30 and its brain tumor-specific module the Quality of Life Questionnaire - Brain Cancer 20 (QLQ-BN20), Functional Assessment of Cancer Therapy-Brain (FACT-Br), and the Short Form-36 Health Survey (SF-36), which all include multiple PRO core constructs (see Table 1) [3].

## SYMPTOMS

### Seizures

In glioma, the mean seizure prevalence is 60%, ranging from 34% in glioblastoma, Isocitrate Dehydrogenase (IDH)-wildtype WHO grade 4, up to 94% in dysembryoblastic neuro-epithelial tumor WHO grade 1, whereas 24% of patients with meningioma and brain metastases experience seizures [11]. Due to the high risk of seizure relapse, the occurrence of a single seizure in patients with a brain tumor is considered epilepsy, requiring treatment with antiseizure medication (ASM) [12^▪▪^]. Adequate seizure management is critical to retain HRQoL, as seizure frequency is a major factor determining HRQoL in patients with epilepsy [13^▪^]. Both clinical decision-making and assessment of seizure outcomes in research is highly based on patient-reported seizure information from patients’ seizure diaries, clinical records, or generic and seizure-specific PROMs [13^▪^]. A useful PROM is the Seizure Severity Questionnaire, which measures the frequency and severity of seizures and their impact on HRQoL [13^▪^]. The QOLIE-31-P, which is designed to measure generic HRQoL in patients with epilepsy, contains six questions on worry, bother and distress related to seizures (see Table 1). The EORTC QLQ-BN20 and MDASI-BT include a single question on seizures, but have limited value as a screening or diagnostic tool for epilepsy due to the short time period covered (i.e., the last week and the last day, respectively). Patient-reported seizure frequency is highly inaccurate due to both over- and underreporting of events as compared to EEG-reporting [14^▪▪^]. However, recent research showed that low self-reported seizure accuracy is sufficient for adequate ASM management [15^▪^]. Patients with an insufficient self-reported seizure accuracy (i.e., < 10% seizure to noise ratio, defined as the sensitivity for true seizures divided by the false alarm rate) may benefit from wearable seizure devices to improve ASM management [16].

### Pain

At diagnosis, around 36% of patients with a brain tumor suffer from headache [17^▪^,18^▪^]. The pathophysiology may involve traction on pain-sensitive structures (e.g., meninges), mass effect from tumor tissue, and cerebral edema [19]. Also, about 13–25% of patients with a primary brain tumor have bodily pain, as compared to 31% of the general cancer population and 55% of patients with advanced or metastatic cancer [18^▪^,^20^]. Apart from traditional nonopioid and opioid analgesic drugs to address cancer pain, cannabinoids are promising in pain management with less adverse effects than opioids, but more research is needed to confirm their efficacy in brain tumor patients [21^▪^]. Multidimensional PROMs instead of unidimensional scales of pain intensity are preferred to assess the experience of pain in brain tumor patients. A commonly used pain-specific measure in patients with cancer is the Brief Pain Inventory covering the intensity, location, and treatment of pain and its interference with other aspects of HRQoL, of which a short form is available [22].

### Difficulty communicating

According to the RANO-PRO Fast Track working group, difficulty communicating is defined as ‘Subjective report of difficulty with the ability to express oneself in speech or writing, or understand speech’^(p. e100)^[4]. It includes aphasia (i.e., impairment to the language function of the brain), dysarthria (i.e., unclear speech due to a pure motor disorder), and speech apraxia (i.e., a higher-level motor planning disorder). Aphasia, either expressive, receptive or mixed, may occur in about 30% of elderly patients with glioblastoma [23^▪^]. It originates from disruptions in the language network, which is a complex neuronal system connecting the Broca region, located in the posterior inferior frontal gyrus, and the Wernicke region, comprising part of the posterior temporal lobe, both in the dominant hemisphere [24^▪▪^]. Importantly, the language network is highly interrelated with cognitive networks, and difficulties communicating and cognitive impairment often coexist [24^▪▪^]. After surgery, patients with tumors in or near eloquent areas are at risk of (transient) speech deficits, especially in case of total resection [25]. As total or supratotal resection is required to maximize survival outcomes, awake surgical resection with intraoperative brain mapping is a well established technique to minimize the risk of language disfunction in patients with brain tumors [26,27^▪^]. Formal tests to objectify language difficulties may not be sensitive enough to identify more subtle patient-reported impairments in language [28]. The QLQ-BN20, FACT-Br, and MDASI-BT all include two or more questions to assess difficulty communicating (see Table 1). Valid HRQoL measures designed for patients with aphasia include the Stroke and Aphasia Quality of Life Scale-39 (SAQOL-39) and the Aphasia Impact Questionnaire (AIQ) – 21, although these have not been validated in patients with brain tumors [29].

### Perceived cognition

Between 46 and 95% of brain tumor patients may have cognitive deficits, depending on the method and timing of measurement [9,17^▪^,30^▪^–32^▪^]. Cognitive deficits are correlated with lower HRQoL and survival [17^▪^,32^▪^]. Tumoral mass-effect, location, and biomolecular characteristics such as isocitrate dehydrogenase (IDH) mutation status in glioma are known determinants of cognitive function [33]. Symptomatic and antitumor treatments, especially whole-brain radiotherapy, have also been associated with cognitive decline, but more research is needed to investigate cognitive toxicity of newer radiotherapy treatment strategies, such as high-precision techniques, tailored dose and fractionation, and proton radiotherapy [8^▪^]. Both PROs and performance outcomes may be valuable and complementary to measure cognition. The most frequently used cognition-specific PROMs are the Medical Outcomes Study Cognitive Functioning Scale (MOS CFS, six items) and the Functional Assessment of Cancer Therapy-Cognitive Function (FACT-cog, 37 items) [3], which cover performance in cognitive domains, such as memory and attention, and its impact on functioning and HRQoL. In a previous report, the RANO group recommended a compact, standardized set of performance outcome measures instead of PROMs as the gold-standard to measure cognition in low-grade glioma patients, due to the higher accuracy of objective testing [34]. However, a recent study found that patients with objective cognitive deficits were sufficiently aware of their impaired cognition to acquire valuable PRO data, although results of PROMs and formal cognitive tests poorly corresponded [35^▪▪^]. As PROMs and performance outcome measures are complementary tools, we recommend to use them in parallel in clinical trials and clinical practice to assess cognitive functioning in brain tumor patients. In patients with cognitive decline, PROs may be substituted by performance outcomes including digit span forward and tests of phonemic and semantic fluency as a minimum set, next to caregiver-reported outcomes [36^▪▪^].

### Symptomatic adverse events

As patients with brain tumors survive longer with more treatment options available, symptomatic adverse effects increasingly hamper their HRQoL. A useful tool to measure patient-reported adverse events of cancer treatments is the National Cancer Institute's Patient Reported Outcome of the Common Toxicity Criteria Adverse Events (NCI PRO-CTCAE) item library, which includes 78 symptomatic adverse events corresponding to the original CTCAE grading system [37]. Efforts have been made to select tumor type-specific item sets from this library, but currently, no set is available for brain tumors yet [37]. The QOLIE-31-P contains four questions on symptomatic adverse events from ASM, which may be used in patients with epilepsy due to a brain tumor (see Table 1). Here, we elaborate on two other major adverse events of brain tumor treatment: cerebral radiation necrosis and fatigue.

Cerebral radiation necrosis is a late complication of radiotherapy, which occurs in about 5–15% of patients, typically 3 months to years after irradiation [38–40]. The pathophysiology involves production of proinflammatory cytokines, which induce fibrinoid necrosis of small vessels thereby causing ischemia and necrosis of brain parenchyma [38,41]. Radiation necrosis may be symptomatic in more than half of the patients, causing worsened neurological symptoms such as seizures [39]. Bevacizumab could effectively reduce radiation necrosis as second-line therapy after corticosteroids and improve seizure control [41,42^▪^].

Despite its multidimensional nature, fatigue was considered a treatment-specific toxicity rather than a core symptom within the PRO priority constructs for high-grade glioma patients [4]. Fatigue is one of the most prevalent symptoms in brain tumor patients, with 57% of glioblastoma patients suffering from fatigue in the early phase [31^▪^]. Symptom network analysis in glioma patients using subscales from PROMs showed that symptoms, especially in fatigued patients, were tightly interrelated [43^▪^]. This illustrates the complexity of targeting fatigue in brain tumor patients, and interventions require a multidimensional approach to increase the success rate. The most frequently used fatigue-specific PROMs in brain tumor patients include the Functional Assessment of Chronic Illness Therapy-Fatigue (FACIT-F), including 13 questions, and the Brief Fatigue Inventory (BFI), which includes three single-item questions and one multi-item question on fatigue and its impact on daily life [3].

## FUNCTIONING

### Physical functioning

All commonly used multidimensional PROMs include items on physical functioning, of which the EORTC QLQ-C30 and SF-36 include separate multi-item scales for physical functioning (see Table 1). Both physical and role and social functioning, as measured with the EORTC QLQ-C30, are determinants of survival in patients with cancer [44]. Patient-reported physical functioning may be impacted during the first phase after diagnosis and treatment of a brain tumor, whereas it may stabilize and improve after years up to decades of follow-up [6^▪^,45^▪^]. Motor rehabilitation is expected to increase physical functioning and decrease fatigue in brain tumor patients [46].

### Role and social functioning

Role and social functioning entail the ability to work or participate in leisure and social activities [4]. Similar to physical functioning, role and social functioning may be most severely compromised in the early phase after treatment in patients with glioma, whereas they may stabilize or improve during long-term follow-up [6^▪^,45^▪^]. Patients with meningioma may have higher role and social functioning compared patients with low-grade glioma, and adjuvant treatment and older age have also been associated with better role and social functioning [6^▪^]. Especially for younger patients with a low-grade brain tumor, such as low-grade glioma or meningioma, returning to work may be an important aspect of role and social functioning and HRQoL. Patients desire to return to work for financial reasons, and they consider it a sign of returning to normality [47^▪^,^48^]. However, about half of meningioma patients may be unable to return to work, and older age and cognitive decline may be determinants of no-return [49]. The QLQ-C30, FACT-Br and FACT-G, and QOLIE-31-P all include separate multi-item scales on role and social functioning (see Table 1). In patients with cognitive decline, the EORTC Instrumental Activities of Daily Living (IADL)- BN32 may be a useful alternative to the QLQ-C30 and QLQ-BN20 to measure daily functioning, both physical and role and social functioning. This questionnaire consists of 32 items and can be filled out by both patients and caregivers separately [50]. The IADL-BN32 has shown acceptable preliminary psychometric properties in a sample of brain tumor patients, and a phase IV validation study is currently being conducted [50].

## PATIENT-REPORTED OUTCOME MEASURES IN CLINICAL TRIALS

Until today, there has been inconsistency in the use of PROs and PROMs in cancer clinical trials [3]. As PROs are gaining a prominent role to inform the benefit-risk assessment of brain tumor treatments, several initiatives have been rolled out to enhance PRO research. In 2018, the Standard Protocol Items: Recommendations for Interventional Trials (SPIRIT)-PRO extension was published [51], which provides a list of items to be included in clinical trial protocols with PROs, such as maximum allowable time windows for PRO assessments [51]. Furthermore, Setting International Standards in Analyzing Patient-Reported Outcomes and Quality of Life Endpoints Data (SISAQOL) initiatives have been established to improve the quality of PRO design, collection, analysis, and interpretation in cancer clinical trials [52,53,54^▪^]. SISAQOL-Innovative Medicines Initiative has agreed on a set of PRO priority objectives, for which recommendations will be developed by the end of 2024 [54^▪^]. Also, PRO item libraries and item banks, which are collections of single items or multi-item scales measuring HRQoL domains of which the latter allow for Computerized Adaptive Testing (CAT), are increasingly available to customize PRO assessment and minimize patient burden [55]. Altogether, these initiatives may help improve the reliability and robustness of PRO data.

## PATIENT-REPORTED OUTCOME MEASURES IN CLINICAL PRACTICE

A key challenge is to apply obtained PRO data in clinical practice so both patients and the treating physicians may benefit from its use. Currently, implementation of PROs in routine oncology and neuro-oncology patient care is still scarce [56,57^▪^]. Oncology and neuro-oncology practitioners recognize the usefulness of PROs, for example to monitor patient symptoms and treatment effect, and to actively involve patients in their own care [58,59^▪^]. Identified barriers include lack of coordination and support from the institution, time constraints, and perceived patient burden or inability [56,58]. However, glioma patients are willing to discuss PROs during follow-up consultations with their healthcare professionals and rate the RANO-PRO priority constructs as important, which supports the feasibility of implementing specific PROs and PROMs in neuro-oncological practice [59^▪^,^60^^▪▪^]. To facilitate this process, an e-learning course is being developed to educate oncological healthcare providers on the use of PROs [61]. For example, after implementation of PROMs in a colorectal surgery clinic, patients completed over 90% of PROMs and surgeons’ review of PROMs in the electronic patient dashboard increased from 7 to 39% during the study period [62]. With the implementation of PROs and PROMs in clinical practice, another challenge will be to guide clinical action in case of concerning PROs to improve health outcomes for individual patients [57^▪^,^61^]. In the future, standardized PRO assessments may even be used as a prognostic tool for ‘objective’ endpoints, as PROs correlate with disease progression and survival in patients with glioma [60^▪▪^].

## CONCLUSION

As ‘quality’ instead of ‘quantity’ of life is increasingly being valued in neuro-oncology, PROs are gaining further importance. Priority constructs of HRQoL recommended for use in clinical trials and practice help to guide clinicians and researchers in selecting meaningful PROMs. While the quality and generalizability of PRO research is improving, there is still work to be done to implement obtained PRO data in clinical practice to benefit patients with a brain tumor. In patients with cognitive impairment, PROs may be complemented or replaced with performance or caregiver-reported outcomes to improve the accuracy of the clinical outcome assessment.

## Acknowledgements

*None.*

### Financial support and sponsorship

*None.*

### Conflicts of interest

*There are no conflicts of interest.*

## REFERENCES AND RECOMMENDED READING

Papers of particular interest, published within the annual period of review, have been highlighted as:

▪ of special interest
▪▪ of outstanding interest
